# Manipulating the NKG2D Receptor-Ligand Axis Using CRISPR: Novel Technologies for Improved Host Immunity

**DOI:** 10.3389/fimmu.2021.712722

**Published:** 2021-08-12

**Authors:** Eric Alves, Emily McLeish, Pilar Blancafort, Jerome D. Coudert, Silvana Gaudieri

**Affiliations:** ^1^School of Human Sciences, The University of Western Australia, Perth, WA, Australia; ^2^Cancer Epigenetics Laboratory, The Harry Perkins Institute of Medical Research, Perth, WA, Australia; ^3^Centre for Molecular Medicine and Innovative Therapeutics, Murdoch University, Perth, WA, Australia; ^4^The Greehey Children’s Cancer Research Institute, The University of Texas Health Science Center at San Antonio, San Antonio, TX, United States; ^5^Perron Institute for Neurological and Translational Science, Perth, WA, Australia; ^6^School of Medicine, University of Notre Dame, Fremantle, WA, Australia; ^7^Institute for Immunology and Infectious Diseases, Murdoch University, Perth, WA, Australia; ^8^Division of Infectious Diseases, Department of Medicine, Vanderbilt University Medical Center, Nashville, TN, United States

**Keywords:** NKG2D, CRISPR, precision medicine, NK cells, viral infection, cancer, immune evasion, immunotherapy

## Abstract

The activating immune receptor natural killer group member D (NKG2D) and its cognate ligands represent a fundamental surveillance system of cellular distress, damage or transformation. Signaling through the NKG2D receptor-ligand axis is critical for early detection of viral infection or oncogenic transformation and the presence of functional NKG2D ligands (NKG2D-L) is associated with tumor rejection and viral clearance. Many viruses and tumors have developed mechanisms to evade NKG2D recognition *via* transcriptional, post-transcriptional or post-translational interference with NKG2D-L, supporting the concept that circumventing immune evasion of the NKG2D receptor-ligand axis may be an attractive therapeutic avenue for antiviral therapy or cancer immunotherapy. To date, the complexity of the NKG2D receptor-ligand axis and the lack of specificity of current NKG2D-targeting therapies has not allowed for the precise manipulation required to optimally harness NKG2D-mediated immunity. However, with the discovery of clustered regularly interspaced short palindromic repeats (CRISPRs) and CRISPR-associated (Cas) proteins, novel opportunities have arisen in the realm of locus-specific gene editing and regulation. Here, we give a brief overview of the NKG2D receptor-ligand axis in humans and discuss the levels at which NKG2D-L are regulated and dysregulated during viral infection and oncogenesis. Moreover, we explore the potential for CRISPR-based technologies to provide novel therapeutic avenues to improve and maximize NKG2D-mediated immunity.

## Introduction

Interactions between hosts and pathogens are constantly evolving, and in this ongoing arms race each side aims to outsmart the other. Host and pathogen genetics form a key part of this competitive evolutionary relationship, with variation in their respective genomes having a considerable impact on host-pathogen dynamics. Many pathogens, such as viruses, generate this variation in the form of mutations as a by-product of their rapid, error-prone replication. Some of these mutations may confer a selective advantage to the pathogen, and *via* the process of natural selection are retained within the variant “pool”, termed quasispecies (1). Interestingly, this process of pathogen evolution bears an uncanny resemblance to what is seen during oncogenesis. During oncogenic transformation, genomic instability gives rise to tumor variants, which undergo a selective process to similarly maintain “fitter” variants within the tumor quasispecies (2). In both the tumor and pathogen contexts, host immune pressure constitutes a major selective force of pathogen/tumor evolution. In humans, the immune response relies on the strategic orchestration of innate and adaptive immunity, which comprises a variety of cell types and soluble molecules. This is regulated by the interaction of multiple receptors and ligands expressed at the cell membrane or released as soluble proteins. As such, pressure exerted *via* receptor-ligand mediated immune responses inadvertently selects for viral or oncogenic mutations that dysregulate receptor/ligand expression (3–5). Consequently, in order to compete against pathogen diversification and oncogenic transformation in this way, humans have over the course of this perennial host-pathogen battle developed in their arsenal a high level of polymorphism at loci encoding receptors/ligands responsible for immune recognition (6–8). One such receptor-ligand axis is the type II lectin-like transmembrane natural killer group 2 member D (NKG2D) receptor and its cognate ligands (NKG2D-L). Herein, we briefly overview the NKG2D receptor-ligand axis in humans, explore the levels at which NKG2D-L regulation/dysregulation occurs, and discuss how clustered regularly interspaced short palindromic repeats (CRISPR)-based technologies are poised to harness NKG2D-mediated immunity in the analogous contexts of oncogenic transformation and viral infection.

## The NKG2D Receptor-Ligand Axis Plays an Important Role in Immune Recognition

### The NKG2D Receptor

**Figure 1:**
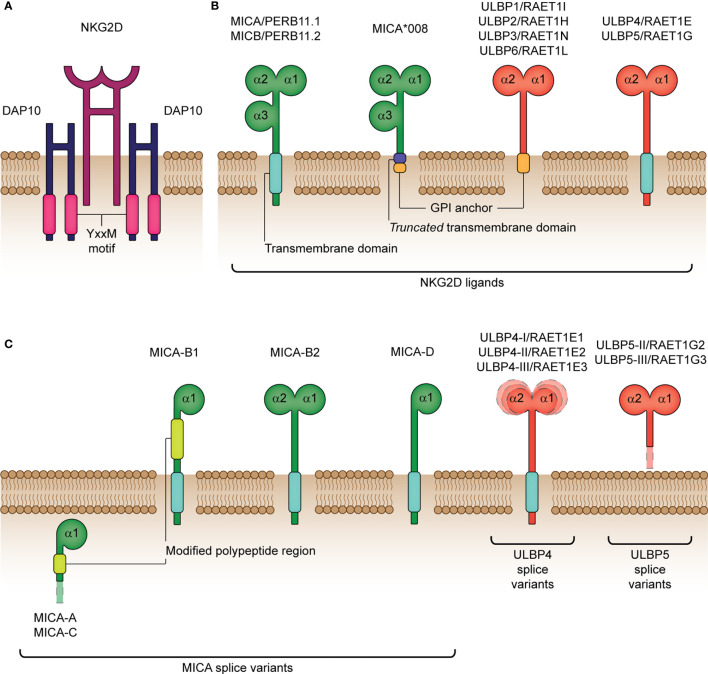
Structure of the human NKG2D receptor and cognate ligands. **(A)** The natural killer group 2 member D (NKG2D) receptor consists of a disulphide-linked homodimer that associates with the DNAX-activating protein 10 (DAP10) disulphide-linked homodimer for cellular signaling. DAP10 harbors a Tyr-X-X-Met (YxxM) motif, which binds the p85 subunit of phosphatidylinositol-3 kinase following phosphorylation. **(B)** NKG2D ligands encompass the MHC class-I polypeptide-related sequence A (MICA), MICB and six UL16-binding proteins (ULBP1-6). MICA/B (also termed PERB11.1/11.2) share similar structural and functional properties, with both containing three extracellular domains (α1, α2 and α3) and a transmembrane domain for binding to the cell surface. In comparison with full-length MICA alleles, MICA*008 differs by encoding a truncated protein due to a nucleotide insertion in the transmembrane domain and is known to acquire a glycolsylphosphatidylinositol (GPI) lipid anchor for cell surface expression. ULBP1-6 (also termed RAET1I/H/N/E/G/L) lack the α3 extracellular domain and are either bound to the cell surface by a GPI-anchor (ULBP1-3, 6) or transmembrane domain (ULBP4, 5). **(C)** Various functional MICA, ULBP4 and ULBP5 splice variants have been identified. MICA-A, -B1, -B2, -C and –D are known isoforms lacking the extracellular α3 domain and the α2 domain in the majority of isoforms (A, B1, C and D). Moreover, MICA-A and MICA-C both lack a transmembrane and cytoplasmic domain, which impairs their expression at the cell surface. RAET1E1/ULBP4-I, RAET1E2/ULBP4-II and RAET1E3/ULBP4-III are membrane-spanning splice variants with an extended α1 domain, reduced α1 domain and reduced α2 domain, respectively. RAET1G2/ULBP5-II and RAET1G3/ULBP5-III are truncated soluble splice variants resulting from two alternative premature stop codons before the transmembrane domain. The surface-expressed splice variants have been shown to bind NKG2D to a similar degree as compared to their wildtype isoforms, except for MICA-B2 and -D that bind NKG2D with a significantly weaker affinity.

NKG2D is the most versatile and widely distributed activating/co-stimulatory natural killer (NK)-related receptor. First identified in human NK cells in 1991 (9), NKG2D has since been discovered on numerous cell subsets including, activated (αβ and γδ) T cells, natural killer T (NKT) cells, and mucosal-associated invariant T (MAIT) cells (10–13). Increasingly, NKG2D expression is also being identified on tissue-resident innate lymphoid cells (ILC), such as certain ILC1 (14, 15), ILC2 (16) and ILC3 (17) subsets. In humans, NKG2D is encoded by the *KLRK1* gene and is located within the NK gene complex (NKC) on chromosome 12p. Moreover, NKG2D is highly conserved across multiple vertebrate species (18, 19). To date, two major human haplotype alleles of NKG2D have been identified, termed *LNK1* (low activity) and *HNK1* (high activity) alleles (20, 21), with surface expression of NKG2D lower in carriers of the low activity *LNK1*/*LNK1* genotype (20, 22). When expressed at the cell surface, the 42 kDa homodimeric NKG2D receptor combines with the DNAX-activating protein 10 (DAP10) homodimer and following its engagement with cognate ligands initiates a cytotoxic cellular response and/or the secretion of pro-inflammatory cytokines (**Figure 1A**) (23–25).

NKG2D is also capable of facilitating the function of other activating receptors, depending on the inflammatory milieu and/or expressing cell type. For example, in activated NK cells primed by pro-inflammatory cytokines (*e.g.* interleukin (IL)-2 and IL-15), NKG2D provides direct stimulatory signals (26–29), whereas in resting NK cells, it synergizes as a co-activator with other receptors, such as NKp46 and 2B4 (30, 31). In αβ T cells, NKG2D typically provides a co-stimulatory signal, acting to promote T cell receptor (TCR)-dependent cytotoxicity, production of pro-inflammatory cytokines and memory differentiation (32–37). Interestingly, prolonged exposure to IL-15 has been shown to increase expression of NKG2D in CD8^+^ T cell subsets, potentiating TCR-independent activation (38, 39). Similarly in γδ T cells, NKG2D can function as a co-stimulatory molecule (40), but may also directly trigger cytotoxicity in a TCR-independent fashion (41). Alternatively, some γδ T cells have been shown to bypass the NKG2D receptor and recognize NKG2D-L, such as ULBP4 or MICA/B, directly *via* their TCR, implying a TCR agonistic role (42–44). In innate-like T cells, such as invariant NKT cells, NKG2D is restricted to the CD4^-^ subsets, and functions to mediate direct lysis of target cells and co-stimulatory activation (12). Whereas, in MAIT cells, NKG2D is more prominent on CD8^+^ subsets, and functions as a co-stimulatory molecule (45) or, if in the presence of IL-15, exerts NKG2D-dependent innate-like cytotoxicity (13, 46). Lastly, in ILC subsets, early studies highlight that expression of NKG2D may aid in the production of pro- or anti-inflammatory mediators depending on the surrounding microenvironment (15, 16). However, further investigation is required to completely elucidate the impact of NKG2D-mediated signaling on ILC function. Altogether, although NKG2D expression, regulation and function differ across the above cell types, it undoubtedly plays a central regulatory role in the immune response and is vital for immunological surveillance against tumorigenic transformation and viral infection.

### The NKG2D Cognate Ligands

Ligands for NKG2D comprise several families of major histocompatibility complex (MHC) class I-related molecules. In humans, these include the MHC class I polypeptide-related sequence A (MICA) and B (MICB), and the human cytomegalovirus (HCMV) glycoprotein UL16-binding protein (ULBP) family (ULBP1-ULBP6) (**Figure 1B**) (47–50). NKG2D-L are, for the most part, not constitutively expressed, but instead are selectively induced upon cellular stress, damage or transformation, as is caused by events such as viral infection or oncogenesis (32, 51). Moreover, as reviewed by Lanier (52), essentially all cell types are capable of expressing one or more types of NKG2D-L if given the appropriate stimulus. For example, Fujita and colleagues (53) identified two distinct ligand expression profiles in non-neoplastic epithelial tissues: ULBP5-ULBP3-MICA/B and ULBP2/6-ULBP1-ULBP4. Moreover, in cells undergoing tumorigenic transformation, high heterogeneity in NKG2D-L expression has been reported. Notably, expression of two or more NKG2D-Ls (often MICA and MICB) is more common in solid tumors, compared to hematological tumors (shown to predominantly express MICB) (54). Furthermore, co-expression of multiple allelic forms of the same ligand has also previously been identified at the surface of cells undergoing stress, which is strongly suggestive of functional redundancy in these molecules. It is also worth noting that NKG2D-L differ in their affinity (*K_D_*) and avidity for NKG2D, such that ULBP1 (the only ULBP member tested to date) has the highest affinity (1.1 μM), followed by MICA (0.9-1 μM) and MICB (800 nM) (55–59). Therefore, it is speculated that NKG2D may transduce different signals or activate separate downstream pathways based on which ligand or allelic variant is bound (60), which supports the various functions of NKG2D discussed prior.

Surprisingly, the role of NKG2D-L extends beyond providing a signal for cellular stress. In cells of the myeloid lineage, these ligands can mediate lymphocyte activation leading to cytotoxicity, cytokine production and proliferation. For instance, expression of MICA/B on dendritic cell-derived exosomes plays an important role in promoting NK cell differentiation and proliferation (61). Furthermore, ULBP2/3 expression levels are increased during CD34^+^ hematopoietic progenitor commitment to the granulomonocyte lineage, suggesting that NKG2D-L play a role in promoting myeloid differentiation (62). Alternatively, NKG2D-L expression on myeloid cells can lead to lymphocyte inactivation and maintenance of immune homeostasis. For example, persistent expression of membrane bound ULBP1 and MICB on myeloid cells induces NKG2D internalization and desensitization of NK cells (63). Moreover, overexpression of MICA on activated CD8^+^ T cells makes them susceptible to NK cell lysis, indicating that NKG2D-L may participate in immune homeostasis during ongoing immune responses (64). During infection with *Mycobacterium tuberculosis*, heightened ULBP1 expression on expanded T regulatory cells (Tregs) facilitates NK cell-mediated killing of these cells, thereby enhancing the overall immune response (65). Ultimately, NKG2D-L expression is associated with both cytotoxic and regulatory processes, as is reflected by the diverse roles played by these molecules in host immunity.

## Redundancy and Overlapping Functions of NKG2D-L Ensure NKG2D Activation

### MICA and MICB

The MICA and MICB genes were originally described as stress-induced MHC class I polypeptide-related sequences and are located in the MHC region on the short arm of chromosome 6 (47, 66). These genes are highly polymorphic (67), with, to date, over 100 described alleles (allelefrequencies.net). The MIC alleles have variations that are, to a certain extent, concentrated in the extracellular domains as well as truncated forms due to coding frame-shifts (*e.g.* MICA 5.1). Specific alleles have been associated with disease outcomes (68–70), influence the amount of soluble protein and impact binding affinity to its cognate receptor, NKG2D (71). Moreover, numerous MICA splice variants have been documented thus far (**Figure 1C**), with the majority binding NKG2D similarly as their wildtype counterparts, highlighting that lack of a domain (*e.g.* α3 domain) does not necessarily reduce binding affinity (60). Interestingly, there is a naturally occurring MICA-MICB null combination (deletion of MICA and premature stop codon for MICB) that exists on the HLA-B48 haplotype found in East Asian and South American populations (72–74). Furthermore, the MICA-MICB genes are merged in chimpanzees resulting in a hybrid form (75), while they are absent in mice (76). The presence of the null haplotype without obvious phenotypic consequences suggests redundancy in the NKG2D receptor-ligand axis but its overall importance is highlighted by the overlapping mechanisms exhibited by cancers and viruses to evade it (77).

### ULBP1-ULBP6

Although the ULBPs are distantly related to MICA/B in sequence, they differ in their location, mapping instead to the opposite (long) arm of chromosome 6 (78, 79). Emerging data suggests that the extensive diversity seen in the ULBP family may be due to the functional or locational specialties of each ligand, as is evidenced with ULBP4 and its predominant expression in skin (80–82). Splice variants exist (ULBP4 (83), and ULBP5 (83, 84); **Figure 1C**) providing significant within locus diversity. Furthermore, as reviewed by Carapito & Bahram (19), clear differences in allele frequencies between geographically distinct populations exist for the ULBP family, which suggest that polymorphisms in ULBP may be a consequence of divergent selective pressures. Moreover, the possession of a large ULBP family in humans and other species is thought to provide a selective host advantage in the evasion of viruses and tumors. However, overall, the ULBP family appears to be less polymorphic than the MIC genes, albeit studies of ULBP gene polymorphisms and haplotypes remain limited (50, 85–88).

## Viruses and Tumors Employ Convergent Mechanisms of NKG2D-L Dysregulation

The appropriate regulation of NKG2D-L is integral to the effective detection and elimination of virally infected or neoplastically-transformed cells. Many reviews to date have discussed the various levels of regulation involved in the control of NKG2D-L expression. For a comprehensive overview of these mechanisms in health and disease, we refer to previously published reviews (59, 89). In this section, however, we focus on convergent regulatory mechanisms exploited by both tumors and viruses to evade NKG2D-mediated immunity.

NKG2D-L expression is regulated at the level of transcription, post-transcription and post-translation through numerous pathways and molecules intrinsically linked to cellular stress (**Table 1**). As such, it is unsurprising that both viruses and tumors harbor various mechanisms that work in combination to hijack and dysregulate NKG2D-L at multiple levels. At the transcriptional level, viral proteins (*e.g.* HBV’s HBx and HBc) have been shown to directly suppress MICA/B (154). A similar strategy is achieved by tumors (*e.g.* melanoma), whereby cells with highly methylated NKG2D-L loci are selected for, given the resultant suppression of transcription (54). Although mechanistically different, both viral proteins and tumor-mediated methylation converge at the DNA level to hinder transcription of NKG2D-L and facilitate immune evasion.

**Table 1:** Established pathways/molecules involved in the regulation of human NKG2D ligand expression and their targetability to date using CRISPR-based genome and epigenome editing.

Level of regulation	Pathway/Molecule	Ligand modulation	Manipulation of pathway/molecule achieved *via* CRISPR genome/epigenome editing	Reference
**Transcription**	Heat shock (*e.g.* HSF1)	↑ MICA/B	No	(42, 90, 91)
		↑ ULBP1/2		
	DNA damage (*e.g.* ATM/ATR)	↑ MICA/B	No	(92–95)
		↑ ULBP1/2/3		
	Oxidative stress (*e.g.* ROS)	↑ MICA/B	No	(96–101)
		↑ ULBP1/2/3/4		
	p53	↑ ULBP1/2	Yes.	(92, 95)
			Achieved: Correction of mutated p53.	
	STAT3	↓ MICA	Yes.	(102, 103)
			Achieved: Genetic deletion of STAT3.	
	NF-κB	↑ MICA	No	(104, 105)
	BCR-ABL	↑ MICA/B	Yes.	(106–109)
			Achieved: Genetic deletion of BCR-ABL.	
	PI3K	↑ MICA/B	Yes.	(110, 111)
			Achieved: Genetic deletion of PI3K.	
	HER2/HER3	↑ MICA/B	Yes.	(110, 112, 113)
			Achieved: Genetic deletion and epigenetic activation/repression of HER2.	
	MAPK	↑ MICA	No	(114, 115)
		↑ ULBP1		
	c-MYC	↕ MICA/B	Yes.	(116–119)
		↕ ULBP1/2/3	Achieved: Epigenetic repression and genetic deletion of c-MYC.	
	TLR-4	↑ MICA	Yes	(120, 121)
			Achieved: Genetic deletion of TLR-4.	
	TLR-7/8	↑ MICA/B	No	(120)
	ATF4	↑ ULBP1	Yes.	(122, 123)
			Achieved: Genetic deletion of ATF4.	
**Post-transcription**	miR-10b	↓ MICB	Yes.	(124, 125)
			Achieved: Genetic deletion of miR-10b.	
	miR-34a/c	↓ ULBP2	No	(126)
	miR-520b	↓ MICA	No	(127)
	miR-17-5p/20a/93/106b/372/373/520c	↓ MICA/B	Yes.	(128–130)
			Achieved: Genetic deletion of miR-93 and epigenetic repression of miR-20a.	
	HCMV-miR-UL112/EBV-pri-miR-BART2-5p/KSHV-miR-K12-7	↓ MICB	No	(131, 132)
	miR-J1-3p	↓ ULBP3	No	(133)
	FUBP3/HuR/XRN2/MATR3/CUGBP1/Vigilin	↓ MICB	No	(134, 135)
	IMP3	↓ MICB	Yes.	(136, 137)
		↓ ULBP2	Achieved: Genetic deletion of IMP3.	
	IGF2BP2	↑ MICB	No	(134)
**Post-translation**	MMP9/MMP14	↓ MICA	Yes.	(138–142)
		↓ ULBP2	Achieved: Genetic deletion of MMP9.	
	ADAM10/ADAM17	↓ MICA/B	Yes.	(143–147)
		↓ ULBP2	Achieved: Genetic deletion of ADAM10 and ADAM17.	
	ADAM9	↓ MICA	Yes. Achieved: Genetic deletion of ADAM9.	(148–150)
	ERP5	↓ MICA	No	(151)
	Histamine	↓ MICA	No	(152)
		↓ ULBP1		
	K5 ubiquitin E3 ligase	↓ MICA/B	No	(153)

↑, Increase; ↓ decrease; ↕ increase or decrease depending on context; CRISPR, clustered regularly interspaced short palindromic repeats; HSF1, heat shock factor 1; MICA/B, MHC class-I polypeptide-related sequence A/B; ULBP1/2/3/4, UL16-binding protein 1/2/3/4; ATM, ataxia-telangiectasia mutated; ATR, ATM and Rad3-related; ROS, reactive oxygen species; STAT3, signal transducer and activator of transcription 3; NF-κB, nuclear factor kappa B; BCR, breakpoint cluster region; PI3K, phosphoinositide 3-kinase; HER/2, human epidermal growth factor receptor 2/3; MAPK, mitogen-activated protein kinase; TLR-4/7/8, toll-like receptor 4/7/8; ATF4, activating transcription factor 4; miR, microRNA; HCMV, human cytomegalovirus; EBV, Epstein-Barr virus; KSHV, Kaposi’s sarcoma-associated herpesvirus; FUBP3, far upstream element binding protein 3; HuR, human antigen R; XRN2, 5’-3’-exoribonuclease 2; MATR3, matrin-3; CUGBP1, CUG triplet repeat RNA binding protein 1; IMP3, IMP U3 small nucleolar ribonucleoprotein 3; IGF2BP2, insulin-like growth factor 2 mRNA-binding protein 2; MMP9/14, matrix metalloproteinase 9/14; ADAM9/10/17, a disintegrin and metalloprotease 9/10/17; ERP5, endoplasmic reticulum protein 5.

At the post-transcriptional level, viruses and tumor cells exhibit convergence in their use of microRNAs (miRNAs) to inhibit NKG2D-L transcript translation. For instance, viral microRNAs have been shown to directly bind to the 3’ untranslated region of MICB (HCMV-miR-UL112, EBV-pri-miR-BART2-5p, KSHV-miR-K12-7) (128, 131) and ULBP3 (JCV-miR-J1-3p) (155), to trigger transcript destabilization and degradation. In addition, viral miRNAs can act indirectly by preventing translation of key components of the GPI-anchoring machinery (HSV1-miR-H8) (156) or blocking translation of surface shedding inhibitors (HCMV-miR-US25-2-3p) (157). Interestingly, host oncogenic miRNAs (124, 126–128, 158) also bind to the 3’ untranslated region of MICA (miR-520b) (127), MICB (miR-17-5p, miR-20a, miR-93, miR-106b, miR-372, miR-373, miR-520c) (128) and ULBP2 (miR-32a/c) (126), in a similar fashion to viral miRNAs, with some binding sites identified by Bauman & Mandelboim (155) as overlapping with those targeted by the viral miRNAs referenced above. Altogether, the shared binding sites and action of oncogenic and viral miRNAs indicate the convergent evolution of tumorigenic and viral immune evasion mechanisms at the post-transcriptional level.

Interference with NKG2D-L expression at the protein level is often a major target of both viruses and tumors. The two major mechanisms of reducing NKG2D-L surface expression have been reviewed by others (77, 89, 159, 160), and are understood to be (1), intracellular retention and degradation, and (2), surface shedding. In the context of viruses, key viral proteins (*e.g.* HCMV’s UL16, UL142) have been found to reside in the endoplasmic reticulum and *cis*-Golgi apparatus of cells, and cause intracellular retention and degradation of MICA/B and ULBP1-3 (161–164). Alternatively, viral infection with HIV or HCMV has also been shown to activate the shedding molecules ADAM10/17, which are otherwise essential for development and homeostasis, resulting in cleavage of MICA/B and ULBP2 (4, 157, 165). Manipulation at the protein level in a similar fashion is also extensively seen in cancer. For example, NKG2D-L have previously been shown to be retained in the endoplasmic reticulum or cytoplasm in a variety of cancer types, including melanoma, breast, colorectal, lung and gastric cancers (51, 166), resulting in reduced cell surface expression. Similarly, NKG2D-L shedding from tumor cells *via* enhanced expression of ADAM10/17, is understood to contribute significantly to the poor immunogenicity of many cancers (143–145). Again, the similarities between viruses and tumors in targeting NKG2D-L at the DNA, RNA and protein level is strongly suggestive of convergent evolution and highlights fundamental immune evasion mechanisms.

## Therapeutic Potential Lies in CRISPR-Mediated Genetic and Epigenetic Manipulation

The ability to modify loci at the genetic and epigenetic level in a specific manner using CRISPR-associated (Cas) proteins, such as Cas9 (167, 168), has greatly expanded our knowledge of diseases, their genetic components and the development of targeted therapies. When combined with a short guide RNA (sgRNA), consisting of a non-coding *trans*-activating RNA annealed to a target-specific 20 nucleotide RNA, Cas9 is able to base pair with any target DNA located adjacent to a conserved protospacer-adjacent motif (5’-NGG-3’ in the frequently used *Streptococcus pyogenes* Cas9) and induce specific DNA cleavage. This process allows efficient and precise DNA editing. Beyond the CRISPR-Cas9 system, the synthesis of engineered variants, such as nuclease-deactivated Cas9 (dCas9), has provided new avenues for gene editing and regulation. The CRISPR-dCas9 system harbors two mutations (D10A and H840A), which deactivate Cas9’s cleavage capability (169). In doing so, the RNA-guided DNA-binding specificity of Cas9 can be harnessed to precisely direct effector domains that mediate transcriptional activation (170–172) or repression (173–175).

Despite the success of wild-type Cas9, its ability to introduce irreversible genetic changes, particularly at off-target sites, has raised safety concerns. Therefore, to date, clinical trials utilizing the CRISPR-Cas9 system have been performed *ex vivo*, where extensive off-target checks can be conducted (176, 177). Although clinical use of Cas9 remains limited due to these trepidations, the application of dCas9 in gene therapy is becoming increasingly likely, given its transient nature and inability to permanently alter the genetic code (178). Moreover, unlike other methods of gene therapy, dCas9-based methods are highly scalable and versatile, with the capability to target multiple loci simultaneously, termed multiplexed editing (179). Furthermore, as seen in combinatorial Cas9 screening systems (180), multiple orthologues of Cas9 can be used concurrently, allowing for synchronized activation and repression of separate loci.

## CRISPR-Based Technologies Offer the Ability to Maximise the NKG2D Pathway in Immunity

In the context of viral infection and cancer, significant potential lies in the use of CRISPR-Cas9 [including other Cas proteins, such as the alternative DNA nuclease Cas12 (181), and RNA nuclease Cas13 (182)] and dCas9-based methods to target NKG2D and its ligands for enhanced immune recognition and elimination. The ease of use, high specificity and multiplexable nature of CRISPR-Cas9/dCas9-based genetic and epigenetic editing has clear applications in the development and improvement of NKG2D-directed therapies, as discussed below.

### CRISPR-Cas9 Genetic Editing

To date, adoptive cell transfer (ACT) therapies, whereby peripheral blood mononuclear cells are collected, edited, expanded and reinfused back into patients, have successfully been developed to express NKG2D (largely on T cells) and shown considerable anti-tumoral and anti-viral potential *in vitro* (183–185). However, *in vivo* responses to NKG2D-focused chimeric antigen receptor (CAR) ACT therapy to date do not appear to be as robust, suggesting that inducing NKG2D alone may not be sufficient (186). To our knowledge, transduction of NKG2D in combination with other receptors within the same cell for improved CAR therapy has only recently been applied clinically, with one (1/9) active registered trial (as of 27 July 2021 on ClinicalTrials.gov) combining transduction of NKG2D and ACE2 for treatment of SARS-CoV-2 (NCT04324996). Most currently active trials (8/9) aim to solely target the NKG2D receptor, and this is partially to reduce the risk of insertional mutagenesis and gene dysregulation (187, 188). CRISPR-Cas9, however, provides a novel avenue for ACT therapy by providing a means to conduct targeted gene insertion in a multiplexed fashion to maximize host immunity (176, 177). Strong evidence suggests that CRISPR-based knockout of receptors responsive to immunosuppressive mediators, such as transforming growth factor beta receptor 2 (TGFβR2), or immune checkpoints (dampeners of cellular activation), such as programmed cell death 1 (PD-1), which play key roles in physiological immune homeostasis, are likely to improve NKG2D-mediated cellular cytotoxicity (189–191). Simultaneous editing of multiple loci, particularly immune checkpoints, in autologous or allogenic cells in this way is also likely to complement immune checkpoint inhibitor (ICI) therapy, such as anti-PD-L1. Moreover, CRISPR-Cas9 expands the potential of ACT therapeutics by facilitating targeted insertion of gene sequences (192), such as *KLRK1*, in cell subsets that otherwise have no or low expression of the NKG2D receptor. Similarly, knockout of inhibitory receptors within the same effector cell using CRISPR-based therapies is predicted to be an excellent starting point in improving ACT therapy outcomes *via* CRISPR-based methods. Notably, enhancement of NKG2D-dependent immune responses in this way requires careful consideration and evaluation of the potential for collateral adverse autoimmune reactions. Indeed, given the central role of the NKG2D receptor-ligand axis in autoimmune conditions, such as Crohn’s disease (36), coeliac disease (193), and rheumatoid arthritis (194), care needs to be taken to not generate autoreactive lymphocytes.

### CRISPR-dCas9 Epigenetic Editing

Apart from direct gene editing to induce or improve NKG2D-mediated immunity, enhancing NKG2D-L on tumor or virus-infected cells represents both an alternative and complementary strategy to augment ACT therapy, ICIs and overall host immunity. In this approach, CRISPR-dCas9-based transcriptional activation or repression may be used to directly activate NKG2D-L loci and ensure their surface expression. Recently, Sekiba et al. (195), have shown this to be possible *in vitro* by applying CRISPR-dCas9 to transcriptionally activate MICA in Huh7 and HepG2 human hepatocellular carcinoma cell lines. Although no other NKG2D-L have been targeted in this way to date, it is predicted that multiplexed activation of several types of NKG2D-L is likely to be most effective in promoting NKG2D-mediated immunity and may resolve the low response rate of NKG2D-ACT therapy *in vivo*. However, our group and others (178, 196, 197) have extensively reviewed that multiplexed CRISPR-based editing, particularly *in vivo* within the tumor or virally infected cell, is best conducted with an optimized set of targets, rather than a large panel, so as to avoid reduced editing efficiency (retroactivity) and extensive off-target effects. Therefore, an accurate understanding of individual ligands and outcome of their binding to NKG2D is strongly recommended. For instance, given the locational and functional specialties within the ULBP family (80, 81), we suggest that activation of these ligands, either individually or in combination, is needed to elucidate their contribution to the NKG2D pathway in different tumoral/viral contexts. Moreover, it is likely that only a subset of ULBP or MIC members need to be targeted, with some unable to engage NKG2D effectively, as previously reported (81). Notably, given the higher affinity of ULBP1 for the NKG2D ligand, compared to MICA and MICB (59), it may serve as a better therapeutic target. However, further investigation is needed to elucidate the affinities of the remaining ULBP family members, and whether higher affinity to the NKG2D ligand directly translates to improved cytotoxicity. CRISPR-dCas9 can also theoretically be applied to other levels of NKG2D-L regulation (**Table 1**) for improved expression and immunity. An obvious example of this is in the transcriptional repression of genes involved in NKG2D-L shedding, such as ADAM10/17 or MMP9/14, which are commonly hijacked during viral infection and tumorigenesis, and are known to be targetable using CRISPR-based technologies (**Table 1**) (143, 165). Targeting the molecules responsible for proteolytic shedding of NKG2D-L in this way is likely to be beneficial both in the clinic and in furthering our basic biological understanding of the mechanisms driving proteolytic shedding. Although CRISPR-dCas9 epigenetic editing is transient and does not induce permanent genetic alterations, significant care needs to be taken to deliver this technology specifically to the target tissue or cell using precise delivery systems, so as to avoid inducing a severe systemic inflammatory state, particularly if used in combination with NKG2D-ACT therapeutics.

## Concluding Remarks

Manipulation of the NKG2D receptor-ligand axis to improve host immunity is emerging as a novel therapeutic avenue in the era of CRISPR-based technologies. In addition to the direct enhancement of NKG2D on cell subsets to generate potent cytotoxic effector cells using Cas9 genetic editing, potential exists to use dCas9-based epigenetic editing methods to activate and lock NKG2D-L expression on tumor or virus-infected cells to promote their recognition and elimination. Although gaps remain in understanding how to optimize NKG2D-mediated immunity in different contexts, CRISPR-based multiplexed editing of NKG2D jointly with other genes on effector cells, or epigenetic activation of NKG2D-L in combination with one another on tumor or virally infected cells is likely to provide important insights to novel therapeutic approaches.

## Data Availability Statement

The original contributions presented in the study are included in the article/supplementary material. Further inquiries can be directed to the corresponding author.

## Author Contributions

Conceptualization, EA and SG. Writing – original draft, EA, EM, JDC, and SG. Writing – review and editing, EA, EM, JDC, SG, and PB. Figures, EA. Funding acquisition, SG. Supervision, JDC and SG. All authors contributed to the article and approved the submitted version.

## Funding

EA is a recipient of an Australian Government Research Training Program Scholarship at The University of Western Australia, BioZone PhD Scholarship at the University of Western Australia and Cancer Council WA PhD Top Up Scholarship. EM is a recipient of an Australian Government Research Training Program Scholarship at Murdoch University. PB is a recipient of an Australian Research Council Future Fellowship (FT130101767), Cancer Council WA Research Fellowship and Wesfarmers Women’s Cancers Fellowship. PB has support from the National Health and Medical Research Council (APP1187328, APP1109428, APP1165208, APP1147528 and APP1130212), the National Institutes of Health (R01CA170370 and R01DA036906), the National Breast Cancer Foundation and Cure Brain Cancer (NBCNBCF19-009), and the US DOD Peer Reviewed Cancer Research Program (CA190006). SG has support from the National Health and Medical Research Council (APP1148284).

## Conflict of Interest

The authors declare that the research was conducted in the absence of any commercial or financial relationships that could be construed as a potential conflict of interest.

## Publisher’s Note

All claims expressed in this article are solely those of the authors and do not necessarily represent those of their affiliated organizations, or those of the publisher, the editors and the reviewers. Any product that may be evaluated in this article, or claim that may be made by its manufacturer, is not guaranteed or endorsed by the publisher.
